# ADHD severity is associated with white matter microstructure in the subgenual cingulum

**DOI:** 10.1016/j.nicl.2015.02.012

**Published:** 2015-02-26

**Authors:** Miriam Cooper, Anita Thapar, Derek K. Jones

**Affiliations:** a Child & Adolescent Psychiatry Section, Institute of Psychological Medicine and Clinical Neurosciences, Cardiff University School of Medicine, Cardiff, UK; b MRC Centre for Neuropsychiatric Genetics and Genomics, Cardiff University School of Medicine, Cardiff, UK; c Cardiff University Brain Research Imaging Centre, Cardiff University School of Psychology, Cardiff, UK; d Neuroscience and Mental Health Research Institute, Cardiff University, Cardiff, UK

**Keywords:** Attention deficit hyperactivity disorder (ADHD), Autism spectrum disorder (ASD), Diffusion MRI, White matter, Spherical deconvolution, Tractography

## Abstract

**Aims:**

This analysis examined hypothesised associations between microstructural attributes in specific white matter (WM) tracts selected *a priori* and measures of clinical variability in adolescents with a diagnosis of attention deficit hyperactivity disorder (ADHD). Firstly, associations were explored between WM microstructure and ADHD severity in the subgenual cingulum. Secondly, to ensure that tract-specific approaches afforded enhanced rather than differential sensitivity, associations were measured between WM microstructure and autistic traits in the right corticospinal tract based on results of a previously-published voxelwise analysis.

**Methods:**

40 right-handed males aged 14–18 years (19 with DSM-IV combined type ADHD and 21 healthy controls) underwent a 60 direction diffusion MRI scan. Clinical ADHD and autism variation were assessed by validated questionnaires. Deterministic tractography based on spherical deconvolution methods was used to map the subgenual cingulum and corticospinal tract.

**Results:**

Fractional anisotropy was positively correlated and radial diffusivity was negatively correlated with a) ADHD severity in the left subgenual cingulum and b) autistic traits in the inferior segment of the right corticospinal tract. No case–control differences were found.

**Conclusions:**

Results shed light on possible anatomical correlates of ADHD severity and autistic symptoms in pathways which may be involved in the ADHD phenotype. They provide further evidence that tract-specific approaches may a) reveal associations between microstructural metrics and indices of phenotypic variability which would not be detected using voxelwise approaches, and b) provide improved rather than differential sensitivity compared to voxelwise approaches.

## Highlights

• Adolescents with ADHD and controls underwent a diffusion MRI scan.
• ADHD severity was associated with microstructure in the left subgenual cingulum.
• Autistic traits in were associated with microstructure in the right corticospinal tract.
• No case–control differences were found.
• Use of tract-specific approaches may improve sensitivity to detect associations.

## 1 Introduction

Attention deficit hyperactivity disorder (ADHD) is a common childhood-onset neurodevelopmental disorder characterised by deficits in the domains of inattention and hyperactivity–impulsivity (Thapar et al., 2012). It is closely associated with autism spectrum disorder (ASD) (Rommelse et al., 2010; Taurines et al., 2012), another developmental disorder which is characterised by social communication deficits and repetitive behaviour. Variation in autistic trait levels provides a major source of heterogeneity in the ADHD phenotype, and have been found to index clinical and cognitive severity in ADHD (Cooper et al., 2014a).

Heterogeneity in imaging findings is analogous to the clinical heterogeneity in ADHD. There is increasing evidence from structural and functional imaging studies of ADHD that aberrant neurodevelopment and brain function is widespread and broadly distributed (Cherkasova and Hechtman, 2009). Using diffusion MRI, cross-sectional ADHD case–control studies have identified spatially widespread and varied alterations in diffusion parameters, which include fractional anisotropy (FA) and mean, axial and radial diffusivities (MD, AD and RD) (van Ewijk et al., 2012). However, identifying specific anatomical areas of relevance to clinical features remains important in order to gain further understanding of the neurobiology of the condition.

Dimensional measures of clinical variability in ADHD (e.g. questionnaire-based measures of ADHD severity and cognitive test measures) have been shown to correlate with white matter (WM) microstructure, but findings here have also been mixed (van Ewijk et al., 2012). Some studies report finding associations between such measures of clinical variability within ADHD and diffusion indices (e.g. Casey et al., 2007; Nagel et al., 2011; Konrad et al., 2010; Konrad et al., 2012; Chuang et al., 2013; Shang et al., 2013; van Ewijk et al., 2014; Wu et al., 2014) but others do not (e.g. Hamilton et al., 2008; Dramsdahl et al., 2012; Silk et al., 2009a,b). However, it is little explored as to whether autistic traits are associated with WM microstructure in ADHD. Only one paper has reported on this to date (Cooper et al., 2014b): in this previous study, in view of the heterogeneity of the results of case–control and correlation analyses to date, we used a non-targeted global voxelwise approach (the widely used pipeline of tract-based spatial statistics (TBSS, Smith et al., 2006)), to test for associations between microstructural metrics and both autistic traits and ADHD symptom severity. Associations were observed between microstructure and autistic trait severity, mostly in the inferior section of the right posterior limb of the internal capsule/corticospinal tract. However no association was found between microstructure and current ADHD severity, in contrast to the results of several previous studies.

Whilst voxel-based global search techniques have their advantages, they do not allow a search to be constrained to a specific anatomical pathway of interest and effectively assume that the properties of neighbouring voxels are independent, even though this will not be the case if they are contained within the same white matter pathway. Diffusion MRI-based tractography (summarised in Jones, 2008) allows three-dimensional reconstruction of WM fibre bundles from diffusion MRI data, permitting integration of diffusion properties along the entire length of specific, anatomically-defined WM pathways. Tractography therefore potentially gives greater power to detect an effect which might be indiscernible using voxelwise approaches.

One methodological challenge is that the diffusion tensor, the model most frequently used to characterise the rate and direction of diffusion in WM, cannot disentangle multiple fibre orientations in a single voxel so cannot delineate complex fibre configurations such as crossing or branching (Alexander, 2005). Tractography based on spherical deconvolution algorithms (such as constrained spherical harmonic deconvolution (Jeurissen et al., 2011; Tournier et al., 2004) and damped Richardson Lucy (Dell'Acqua et al., 2010)) provides a more accurate method of detailing the underlying architecture of specific fibre bundles, thus allowing their possible role in the pathophysiology of a clinical disorder to be explored more reliably.

In this paper we focused specifically on a targeted search of an area of interest using a tract-specific rather than a global approach, in the same sample that we had previously analysed using TBSS. We focused our attention entirely on the subgenual cingulum. The cingulum is the major WM pathway connecting to the cingulate cortex, whose anterior portion is an area which has been repeatedly implicated as abnormal in ADHD, in terms of smaller volumes (e.g. Seidman et al., 2006; Amico et al., 2011; Makris et al., 2010), slower rate of thinning in adolescence (e.g. Shaw et al., 2011), decreased activity on task-based fMRI (e.g. Bush et al., 1999; Smith et al., 2008), and decreased functional connectivity (e.g. Qiu et al., 2012). The cingulum has been found to have diffusion abnormalities in ADHD in adults (Konrad et al., 2010; Makris et al., 2008). It has been found that the cingulum may be better represented by division into three distinct subcomponents (subgenual, retrosplenial and parahippocampal) with differing diffusion properties as opposed to one homogeneous fronto-temporal association tract (Jones et al., 2013). The subgenual cingulum is the most rostral section and the pathway located most proximally to the anterior cingulate.

We tested the very specific anatomical hypothesis that variation in microstructure throughout the whole subgenual cingulum would be associated with ADHD severity, and that the use of tractography would enhance statistical power to detect an effect which had not been evident in the voxelwise analysis. In addition, we aimed to ensure that tractography affords enhanced, rather than differential, sensitivity as compared to voxelwise approaches. We therefore also re-examined the relationship between autistic trait scores and microstructural metrics in the right corticospinal tract that we previously found using TBSS, to confirm that increasing autistic trait scores would be positively correlated with FA and negatively correlated with RD (Cooper et al., 2014b). Case-control analyses were also undertaken for the subgenual cingulum and the right corticospinal tract.

## 2 Methods

Ethical approvals, sample recruitment, consent and clinical characteristics are described in detail elsewhere (Cooper et al., 2014b). Forty right handed male participants (19 clinic patients with DSM-IV combined type ADHD, 21 controls) were recruited. Diagnosis of ADHD had been confirmed by a research diagnostic interview, the Child and Adolescent Psychiatric Assessment (CAPA, Angold et al., 1995). Individuals were required to be aged 14–18 years at the point of scanning, and have IQ test scores of >70 as ascertained by WISC-IV (Wechsler, 2003).

Demographic information and trait measures of psychopathology were assessed by questionnaires that were completed by the parent. Current ADHD severity was ascertained by a modified version of the DuPaul ADHD scale (DuPaul, 1991, scoring described in Cooper et al., 2014b), a validated measure of ADHD severity. Autistic traits were assessed using the Social Communication Questionnaire (SCQ, Berument et al., 1999). Overall burden of current psychopathology was measured using the Strengths and Difficulties Questionnaire (SDQ, Goodman, 1997).

After being settled into the process using a ‘mock’ scanner, subjects underwent a 3 T MRI (GE Signa HDx) scan with diffusion encoded along 60 non-collinear optimally ordered directions (Cook et al., 2007; Jones et al., 1999) with a *b* value of 1200 s/mm^2^, and six initial scans of *b* = 0 s/mm^2^. Peripheral gating to the cardiac cycle was used during data acquisition.

ExploreDTI version 4.8.2 (Leemans et al., 2009) was used for all diffusion data processing. Correction for subject motion and eddy current distortion was applied (Haselgrove and Moore, 1996), as was reorientation of the encoding vectors to allow for subject rotation (Leemans and Jones, 2009). Non-iterative weighted linear least squares regression was then used to fit a tensor model to each voxel (Basser et al., 1994). Residuals to the tensor fit were inspected for outlying data points (Tournier et al., 2011), and those with significant artefact on their scans were omitted from all analyses (*n* = 2 from each group).

Whole brain deterministic tractography using the damped Richardson Lucy algorithm (dRL, Dell'Acqua et al., 2010), was implemented on the diffusion data. dRL reduces false positives over other SD methods, whilst maintaining angular resolution (Dell'Acqua et al., 2010), providing the anisotropy of the ideal fibre response is relatively high (Parker et al., 2013). Seedpoint resolution was 2 × 2 × 2 mm, the step size was 0.5 mm, *L*_max._ was 8 and tracking was terminated when the angle threshold of the pathway changed through >45°. Regions of interest (ROIs) based on Boolean logical operations (SEED, AND and NOT gates) were drawn to define the subgenual cingulum bilaterally and the right corticospinal tract, which were drawn in each subject's native space on directionally encoded colour maps based on the principal eigenvector of the diffusion tensor. Fibres of the contralateral hemisphere were excluded by the placement of a NOT gate in the sagittal plane two slices (3.75 mm) lateral to the mid-sagittal plane in the hemisphere that was not of interest. Once tracts were drawn, fibres inconsistent with known anatomy were excluded by NOT gates. The corticospinal tract was split into superior and inferior segments on the basis of the previous TBSS results (Cooper et al., 2014b). The protocols for tract reconstructions are detailed in the Appendix.

The Robust Estimation of the Tensor by Outlier Rejection (RESTORE) algorithm (Chang et al., 2005), which aims to correct for data artefacts arising as a consequence of physiological noise such as subject motion and cardiac pulsation, was then used to derive FA and MD values from the tracts drawn in the dRL data. For all analyses, where significant results (*p* < 0.05) were seen for FA or MD, AD and RD were explored to further investigate the nature of these results.

Analyses were carried out in SPSS, version 18.0 (SPSS Inc., 2009). Group differences in the distributions of demographic and clinical variables were analysed by Mann–Whitney *U* tests.

Shapiro–Wilk tests were used to check normality (Shapiro–Wilk statistic >0.05) of the distributions of total ADHD/SCQ scores in the ADHD group. Total ADHD scores were normally distributed but total SCQ scores were not. Total SCQ scores were transformed to normality using ln(score + 1).

For each tract, the distributions of FA and MD (and where indicated, AD and RD) were also checked for normality (Shapiro–Wilk statistic >0.05). The majority were normally distributed. Those which were not (FA in the right subgenual cingulum in both ADHD and control groups, MD in the right inferior corticospinal tract in the ADHD group) were not amenable to transformation.

For the correlation analyses, where both symptom scores and diffusion parameters were normally distributed, Pearson's coefficient was used, otherwise, Spearman's coefficient was used. Correlations were tested between diffusion parameters and the following phenotypic variables: a) total ADHD score, in the subgenual cingulum bilaterally and b) SCQ score, in the right corticospinal tract (including superior and inferior sections).

Logistic regressions were used to test for case–control differences in microstructure, with case/control status as the outcome and diffusion parameters as the predictor variables. Regressions were then re-run with age as a covariate. As logistic regressions do not have a formal requirement for normality of the independent variable (Spicer, 2004), regressions were run for all diffusion parameters of the tracts under study.

## 3 Results

Two participants were excluded from each group due to scan artefact, leaving 17 in the ADHD group and 19 in the control group. Therefore correlation analyses in the ADHD group were carried out with *n* = 17. Two of the remaining controls had high reported levels of inattentive/hyperactive–impulsive symptoms and were thus excluded from the case–control analyses, meaning these were run with 17 ADHD and 17 controls. Two participants in the ADHD group had low endorsement of current ADHD symptom scores, they were considered ‘remitted’ and the case–control analyses were subsequently re-run with them excluded i.e. with 15 ADHD and 17 controls.

Table 1 shows clinical and demographic characteristics of those in the main analyses (*n* = 17 ADHD and *n* = 17 controls), and Mann Whitney *U* test statistic results for differences in the distributions of these variables. In both the ADHD and control groups, neither total ADHD nor SCQ scores were correlated with age. All controls had an SDQ total difficulties score within in the normal range and none had any SDQ subscale score in the abnormal range. 16/17 of the ADHD group had a medication history, of treatment with short/long acting stimulants and/or atomoxetine. Six of these had discontinued their medication prior to the scan.

**Fig. 1:**
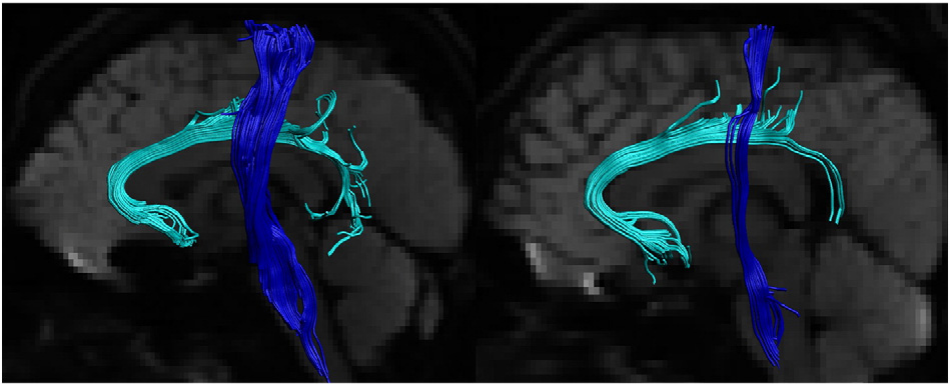
Tractography reconstructions from two individuals. Tractography reconstructions from two individual (left side shown). Light blue — subgenual cingulum. Blue — corticospinal tract. Tracts overlain on mean of diffusion weighted images. The CST was split into inferior and superior segments at the level of the lateral sulcus.

Examples of tractography reconstructions are shown in Fig. 1.

**Fig. 2:**
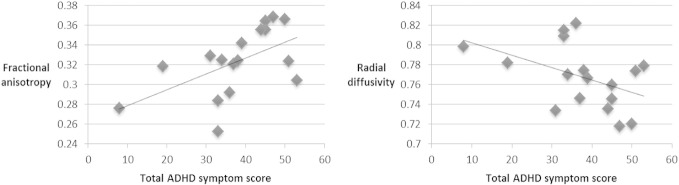
Scatter plot of correlations between diffusion parameters and total ADHD symptom scores in the left subgenual cingulum. Units for radial diffusivity = 10^−3^ mm^2^ s^−1^. Linear trendlines are shown. For fractional anisotropy, *R* = 0.553 (*p* = 0.021). For radial diffusivity, *R* = −0.454 (*p* = 0.067).

Significant positive correlation was found between current ADHD severity (indexed by total ADHD score) and FA in the left subgenual cingulum (Pearson's coefficient 0.553, *p* = 0.021), accompanied by trend significant negative correlation with RD (Pearson's coefficient −0.454, *p* = 0.067) (Table 2, Fig. 2). No associations with FA (*p* = 0.859) or MD (*p* = 0.704) were found in the right subgenual cingulum.

**Fig. 3:**
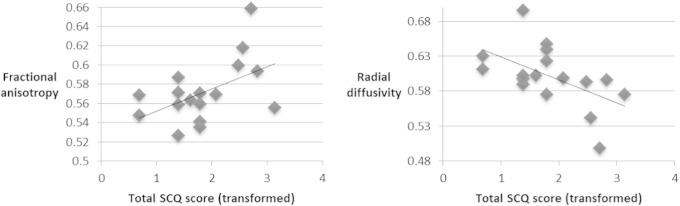
Scatter plot of correlations between diffusion parameters and autistic symptom scores in the right inferior corticospinal tract. SCQ, social communication questionnaire. Units for radial diffusivity = 10^−3^ mm^2^ s^−1^. Linear trendlines are shown. For fractional anisotropy, *R* = 0.506 (*p* = 0.038). For radial diffusivity, *R* = −0.542 (*p* = 0.025).

A weak trend towards association was seen between autistic trait score and FA (Pearson's coefficient 0.358, *p* = 0.158) and autistic trait score and MD (Pearson's coefficient −0.401, *p* = 0.111) in the right corticospinal tract. When this tract was divided into inferior and superior segments, significant associations were found in the inferior corticospinal tract only, for FA (Pearson's coefficient 0.506, *p* = 0.038) and RD (Pearson's coefficient −0.542, *p* = 0.025) (Table 3, Fig. 3). No significant associations were found in the superior segment for either FA (*p* = 0.658) or MD (*p* = 0.183).

In line with the previous global voxelwise analysis of the same subjects, no case–control differences in FA or MD were found for the subgenual cingulum or total right corticospinal tract, and no case–control differences were revealed with the addition of age as a covariate (*n* = 17/17). Results were unchanged with the exclusion of the two ADHD subjects considered as remitted (*n* = 15/17).

## 4 Discussion

As hypothesised, associations were found between WM microstructure and current ADHD severity in the subgenual cingulum (left), which had not been evident in the previous voxelwise analysis of the same sample of young people with ADHD. Also, the same pattern of microstructure (increased FA, decreased RD) was found to be associated with increasing autistic traits in the right inferior corticospinal tract as it was in the voxelwise analysis, confirming that tractography does indeed provide enhanced, rather than differential, sensitivity. Results based on anatomically-defined reconstructions of specific tracts potentially have more biological plausibility: in neurodevelopmental disorders, it is more likely that an entire pathway will be affected rather than just an isolated piece of tissue contained in a voxel. These analyses highlight pathways which merit further investigation in future research into clinical variability of the ADHD phenotype.

The associations between WM microstructure and ADHD severity in the subgenual cingulum are in keeping with previous literature implicating both the anterior cingulate and the cingulum in the pathophysiology of ADHD. However these results are the first to demonstrate correlation between microstructure and ADHD symptom severity specifically in the anterior (subgenual) portion of the cingulum, which directly underlies the anterior cingulate. The fact that the association was seen in the left hemisphere is at odds with a meta-analysis of fMRI studies which found that the right rather than the left anterior cingulate cortex was one of the areas with dysfunction during tasks of inhibition (Hart et al., 2013), although exactly how alterations in diffusion metrics relate to cortical activity is not yet clear (Jones, 2010). However, there is evidence that individual differences in the microstructure of the left anterior segment of the cingulum may be related to individual differences in cognitive control, including attention switching and response inhibition (Metzler-Baddeley et al., 2012). That this present association was found using tractography but was not seen in the TBSS analysis in the same sample may well be due to the increased anatomical specificity and the greater statistical power to detect associations afforded by tractography as compared to a voxelwise approach, and similar effects have been previously reported (Keedwell et al., 2012).

The associations between autistic traits and WM microstructure in the right corticospinal tract support the TBSS findings from the same sample (Cooper et al., 2014b), confirming localisation of the association to the inferior segment. It is worth noting that associations between autistic traits and microstructure in the right corticospinal tract in its entirety did not reach statistical significance and it was only when the tract was segmented into superior and inferior that the significant effect was located in the inferior region, analogous to the TBSS results. It is possible that the exact architecture of a tract may impact on the sensitivity to detect an effect at a particular point. The corticospinal tract bundle ‘funnels’ down to become progressively more compact from its superior regions, where fibres fan out somewhat, to its inferior regions, where fibres are more closely aligned. It may be that in the inferior region where intravoxel orientational coherence is high, measures such as the anisotropy will have more sensitivity to changes in the microstructure of individual axons compared to superior regions where orientational dispersion is the main factor influencing anisotropy.

Total ADHD/ASD scores and FA were positively correlated (i.e. increased symptom severity was associated with increased FA). Total ASD score was negatively correlated with RD, and there was a trend towards a negative association between total ADHD score and RD. Although ADHD case status and increasing symptom severity have tended to be associated with decreased FA (e.g. Ashtari et al., 2005; Hamilton et al., 2008; Makris et al., 2008; Nagel et al., 2011; Dramsdahl et al., 2012; Chuang et al., 2013), there is variability in the literature and some studies do report the opposite effect (e.g Silk et al., 2009b; Davenport et al., 2010; Li et al., 2010; Peterson et al., 2011). However, a recent large study has reported ADHD status to be associated with decreased FA and decreased MD compared to controls, but has reported increasing ADHD symptom severity to be associated with *increased* FA and decreasing MD (van Ewijk et al., 2014), and the authors suggest that different mechanisms may underlie ADHD diagnosis versus heterogeneity within ADHD, at least with regard to the diffusion literature. However, there is also variability in both the ADHD and ASD diffusion literature to date in terms of characteristics which could potentially be confounding observed associations due to their independent associations with microstructure, including gender, handedness and the inclusion of wide age ranges (reviewed in van Ewijk et al., 2012 and Travers et al., 2012). The use of an SD algorithm, which gives greater fidelity than tensor based tracking approaches, combined with the use of a high angular resolution (60 direction) cardiac-gated acquisition sequence and the RESTORE algorithm to maximise accuracy in estimation of the tensor may have the potential to characterise alterations in diffusion parameters more robustly than earlier diffusion imaging methods. However, the exact neurobiology (e.g. axonal diameter, density, branching and myelination) underlying the observed alterations in microstructure cannot be characterised conclusively without advanced neuroimaging techniques such as composite hindered and restricted model of diffusion (CHARMED, Assaf et al., 2004) magnetisation transfer ratio (MTR, Morrison and Henkelman, 1995), quantitative magnetisation transfer imaging (qMT, Sled and Pike, 2001) and multicomponent relaxometry (e.g. Deoni et al., 2008). However, it can be hypothesised that the increase in FA and decrease in RD associated with increased neurodevelopmental symptom burden in in the present analyses may be ascribable to one or more of a) decreased neural branching b) denser packing of fibres, or c) increased myelination. Further work would be needed to confirm this.

In line with the previous TBSS findings from this sample, no case-control differences in microstructure were found in the tracts examined, despite the use of rigorous methodology to examine microstructure in specific tracts and the increased power to detect effects afforded by an extended ROI approach i.e. the tractography method reported here. It is not possible to rule this out as being an effect of medication. It remains unclear as to whether stimulants and atomoxetine may have a role in normalising microstructure as studies have not yet been set up to investigate this question explicitly. However there is evidence to suggest that stimulants may attenuate alterations in a) macrostructure (Castellanos et al., 2002; Nakao et al., 2011), b) brain function (e.g. Rubia et al., 2009) and the trajectory of cortical development (Shaw et al., 2009). At first glance the positive associations between FA and ADHD/ASD trait scores in the present study may seem at odds with the lack of case–control differences in the same areas. However, it is possible to envisage scenarios in which the means of FA in each group are equal (i.e. no case–control difference exists) but in which the data in each group have a different spread about the mean (reflected in the correlation). Thus case–control difference and trait score versus FA correlation need not be coupled.

Strengths of these analyses are as follows. The ADHD group was a thoroughly assessed clinical group, all with a lifetime diagnosis of DSM-IV ADHD-C to decrease heterogeneity in the phenotype. All were right-handed males to remove the confounding influence of gender and handedness. The age range included only older adolescents to try to minimise the effect of age on brain structure. The lack of a prerequisite to stop medication will have helped even out group differences in movement during scans. The combination of 60 direction diffusion imaging, cardiac gating and the RESTORE algorithm will have improved robustness in the estimates of diffusion parameters. The use of an SD algorithm will have improved specificity of fibre tracking compared to models based on the tensor alone.

Limitations are as follows. There was a statistically significant group difference in the distribution of age, and although age was not found to exert any influence as a covariate in the case–control analyses, this difference should be considered a limitation to the group comparisons. Many of the correlations would not have survived a correction for multiple testing, but might have been of greater strength in a larger sample. Nonetheless, the risk of results being ascribable to type 1 errors was reduced by adopting a hypothesis-driven approach with regards to the few tracts under study. By virtue of how they are calculated, the diffusivity measures reported here are not mutually independent of each other as they are all computed from the three eigenvalues of the diffusion tensor. Thus it was not appropriate to apply a multiple testing correction in these regards. Data are cross-sectional so no inference can be made about direction of effects or causality. Also, whilst SD methods afford better anatomical specificity and veracity of pathway reconstruction over tenser-based tracking approaches, they cannot in themselves improve quantification of the underlying microstructural characteristics (i.e. FA, MD, AD and RD). These tenser-based parameters, which are projected onto the SD-derived tracts, will still be subject to the tensor's inability to resolve complex architecture. This problem could be resolved, for example, by using parameters derived directly from the fibre orientation distribution function, such as the hindrance modulated orientational anisotropy (HMOA, Dell'Acqua et al., 2013) or the apparent fibre density (AFD, Raffelt et al., 2012). The development and implementation of these parameters is the focus of continuing research. Caution should also be taken when adopting a hypothesis driven, region-of-interest approach to WM analysis in view of diffusion covariance: indices of microstructure may be correlated throughout the brain within an individual, meaning that positive findings may not be confined to the investigated area alone and caution should be exercised when localising an explanatory effect to the investigated region only.

In summary, this study investigated associations between ADHD severity and autistic traits and WM microstructure in ADHD, using tractography in combination with SD methods to help improve the anatomical fidelity of pathway reconstruction through areas of complex fibre architecture. Results suggest that certain patterns of WM microstructure are correlates of ADHD severity and autistic traits and provide clues on pathways to investigate further in future research. They also highlight the benefit of adopting tract-specific approaches to improve power to detect effects, and suggest that such approaches indeed provide improved rather than differential sensitivity.

## Conflict of interest

The authors have no conflicts of interest to report.

## Figures and Tables

**Fig. 4:**
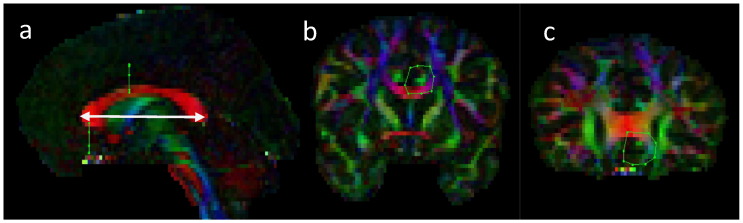
ROI placement for the subgenual cingulum. ROI placement for subgenual cingulum (left hemisphere): a) midsagittal plane — location of both ROIs, b) coronal plane — AND gate five slices anterior to the midpoint of the corpus callosum, c) coronal plane — AND gate three slices caudal to the anterior limit of the genu of the corpus callosum.

**Fig. 5:**
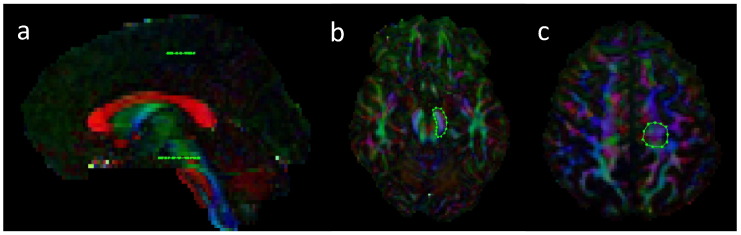
ROI placement for the corticospinal tract. ROI placement for the corticospinal tract (left hemisphere): a) sagittal view — both ROIs, b) axial plane — AND gate round the cerebral peduncle at the level of the decussation of the superior cerebellar peduncle, c) axial plane — AND gate round the motor cortex. The tract was split into inferior and superior segments at the level of the lateral sulcus.

**Table 1:** Demographic and clinical sample characteristics.

		ADHD group (n = 17)		Control group (n = 17)		U value	*p* value
		Mean	SD, range	Mean	SD, range		
Age, years		15.6	(1.3, 14.3–18.6)	16.9	(1.2, 15.0–18.8)	223	0.006
Mean full scale IQ		87.6	(9.8, 75–110)	106.9	(7.6, 98–122)	265	<0.001
Mean total ADHD symptoms score (max 54)		37.8	(11.5, 8–53)	4.5	(4.2, 0–14)	4	<0.001
Mean SCQ score (max 40)		7.1	(5.90, 1–22)	1.0	(1.06, 0–3)	19	<0.001
Mean SDQ difficulties score (max 40)		19.1	(8.00, 5–35)	2.9	(2.45, 0–8)	285	<0.001
Family income group^a^	Low, %	50		5.9		252	<0.001
Medium, %	50		11.8	
High, %	0		82.4	
Took medication on morning of scan day	Yes	10		N/a		N/a	N/a
No	6		N/a		N/a	N/a
Unknown	1		N/a		N/a	N/a

HI, hyperactive–inattentive; I, inattentive; SCQ, Social Communication Questionnaire; SDQ, Strengths and Difficulties Questionnaire. U value = Mann Whitney U test statistic for differences in the distribution of variables between groups.

a Low: annual household income <£20,000, medium: annual household income £20–40,000, high: annual household income >£40,000 (information not available for one participant with ADHD).

**Table 2:** Correlations between diffusion parameters and ADHD symptom scores in the left subgenual cingulum.

	Total ADHD score
Left SGC: FA	0.553 (*p* = 0.021)**
Left SGC: MD	−0.248 (*p* = 0.338)
Left SGC: RD	−0.454 (*p* = 0.067)^a^
Left SGC: AD	0.318 (*p* = 0.214)

All figures are Pearson's coefficient. SGC — subgenual cingulum. FA — fractional anisotropy. MD — mean diffusivity. RD — radial diffusivity. AD — axial diffusivity.

** Significant at *p* < 0.05 (2-tailed).

a Trend significant.

**Table 3:** Significant correlations between diffusion parameters and ASD symptom scores.

	Total SCQ score
Right inferior CST: FA	0.506 (*p* = 0.038)**
Right inferior CST: MD	−0.395 (*p* = 0.117)^a^
Right inferior CST: RD	−0.542 (*p* = 0.025)**
Right inferior CST: AD	0.120 (*p* = 0.647)

All other figures are Pearson's coefficient. Italics — post-hoc tests of correlations with subscale scores. SCQ — social communication questionnaire. CST — corticospinal tract. FA — fractional anisotropy. MD — mean diffusivity. RD — radial diffusivity. AD — axial diffusivity.

** Significant at *p* < 0.05 (2-tailed).

a Spearman's rho.

## Appendix

The subgenual cingulum was reconstructed based on the method of Jones et al. (2013). The mid-sagittal plane was located, and on this, the rostro-caudal midpoint of the corpus callosum was identified as the mid-point between the anterior limit of the flexure of the genu, and the posterior co-ordinate of the flexure of the splenium. Two AND gates were then drawn around the location of the cingulum in the coronal plane (Fig. 4a, b, c): the first, five slices anterior to the midpoint of the corpus callosum and the second, three slices caudal to the anterior limit of the genu. Tracking was then initiated.

The corticospinal tract was drawn, between the midbrain and the primary motor cortex, based on the methods of Wakana et al. (2007). In the axial plane, an AND gate was placed around the cerebral peduncle at the level of the decussation of the superior cerebellar peduncle and another AND gate was placed around the motor cortex, which was identified anterior to the central sulcus (Fig. 5a, b, c). The CST was then split into inferior and superior segments at the level of the lateral sulcus.
